# Nordic survey on practice of neurosurgical management of craniopharyngioma in children

**DOI:** 10.1007/s00381-026-07153-8

**Published:** 2026-02-04

**Authors:** Radek Frič, Anh-Thu Nora Tran, Bernt Johan Due-Tønnessen, Daniel Nilsson, Jon Foss-Skiftesvik

**Affiliations:** 1 Department of Neurosurgery, Oslo University Hospital–Rikshospitalet, P.O.Box 4950, N-0424 Oslo, Norway; 2 Pediatric Neurosurgical Research Group, Faculty of Medicine, University of Oslo, Oslo, Norway; 3 Faculty of Medicine, University of Oslo, Oslo, Norway; 4 Department of Neurosurgery, Sahlgrenska University Hospital, Gothenburg, Sweden; 5 Department of Neurosurgery, Rigshospitalet University Hospital, Copenhagen, Denmark

**Keywords:** Pediatric craniopharyngioma, Management, Surgical treatment

## Abstract

**Purpose:**

Management of childhood-onset craniopharyngioma (CP) may be challenging, given its rarity and complexity as well as the risks associated with treatment. We aimed at investigating how the practice of neurosurgical management of CP in children differs among neurosurgical centres in Nordic countries.

**Methods:**

We performed a survey containing 28 questions and focusing on organization of care, expertise availability, treatment decisions and strategies, and follow-up routines, respectively. The survey was distributed to 21 neurosurgical centres in five Nordic countries.

**Results:**

We obtained answers from 18 centres (86% response rate). In 61% of centres, only 0–1 new cases are treated per year. In only one country (Denmark), the neurosurgical treatment of pediatric CP is centralized to one centre. While all cases are discussed at MDT meetings before surgery and during the follow-up, management strategies, including surgical indications, goals, and approaches, vary substantially among responding centres.

**Conclusion:**

The practice of neurosurgical management of pediatric CP in the Nordic countries is characterized by relatively low case volumes, differing management strategies, as well as varying degrees of centralization and collaboration between pediatric and adult neurosurgeons. Although some practical aspects may be unique for healthcare practised in the Nordic countries, the results of this survey and, in particular, the embedded discussion of centralization – either restricted to surgery or including complete management of pediatric CP – seem relevant and may provide useful insights also to neurosurgeons outside of the Nordics.

**Supplementary Information:**

The online version contains supplementary material available at 10.1007/s00381-026-07153-8.

## Introduction

Craniopharyngiomas (CP) are rare, benign tumours encountered in both children and adults, yet with a tendency to aggressive growth into critical neural structures. This feature, along with significant morbidity associated with treatment, makes the choice of optimal treatment strategy difficult, particularly in children.

To reduce the morbidity related to treatment and specifically to radical surgical resections [1–3], there has been a tendency towards less aggressive surgical approaches in children with CP during the recent two decades [4–6]. Yet, the practice of treatment still differs among centres, which may lead to some controversies [7, 8]. Moreover, there are significant differences among countries regarding the availability and practice of specialized health care. All these differences may significantly confound the comparison of treatment outcomes in pediatric CP series.

The purpose of this survey is to gain an overview of the current standard of practice in neurosurgical management of children with CP across five Nordic countries (Denmark, Finland, Iceland, Norway, Sweden). The public healthcare systems in all these countries are very similar, characterized by universal access, unique patient identification numbers, and high-quality national health registries. Treatment of difficult pathologies like CP occurs at a relatively few neurosurgical centres. Together with stable populations and generally good follow-up routines in all included countries, this allows for a relevant comparison of treatment strategies between centres.

Given the rarity of the CP, with 0.5–2.5 new cases per 1.000.000 inhabitants a year, and approximately half of it children [9], we also wondered how the limited occurrence in Nordic countries, given their relatively small populations in general (combined population of all these five countries being currently approximately 28.3 millions), affects decision making among neurosurgeons in these countries.

## Methods

During a few recent years, an informal forum of Nordic pediatric neurosurgeons has been established—Nordic Pediatric Neurosurgical Network – for exchange of ideas, experience, and coordination of collaborative efforts. Following discussions regarding challenges in the treatment of pediatric CP in this forum’s meeting in September 2024, the idea of a survey on practice emerged. The survey was then elaborated within a working group consisting of four pediatric neurosurgeons (R.F., D.N., J.F.-S., B.J.D-T.) from three different departments and countries.

The survey (supplementary material) was written in English and consisted of 28 questions, divided into three main areas: A. Organization and expertise, B. Treatment decision and strategies, and C. Follow-up routines. Upon circulation of the survey, we asked one principal representative from each department (typically a leading pediatric neurosurgeon) to complete the survey on behalf of his/her institution.

The information provided was treated confidentially and anonymized, stored in a dedicated data file, where no responses could be tracked back to specific institutions. An independent observer (A.N.T.), not participating in the preparation of the survey, summarized the replies.

## Results

**Fig. 1:**
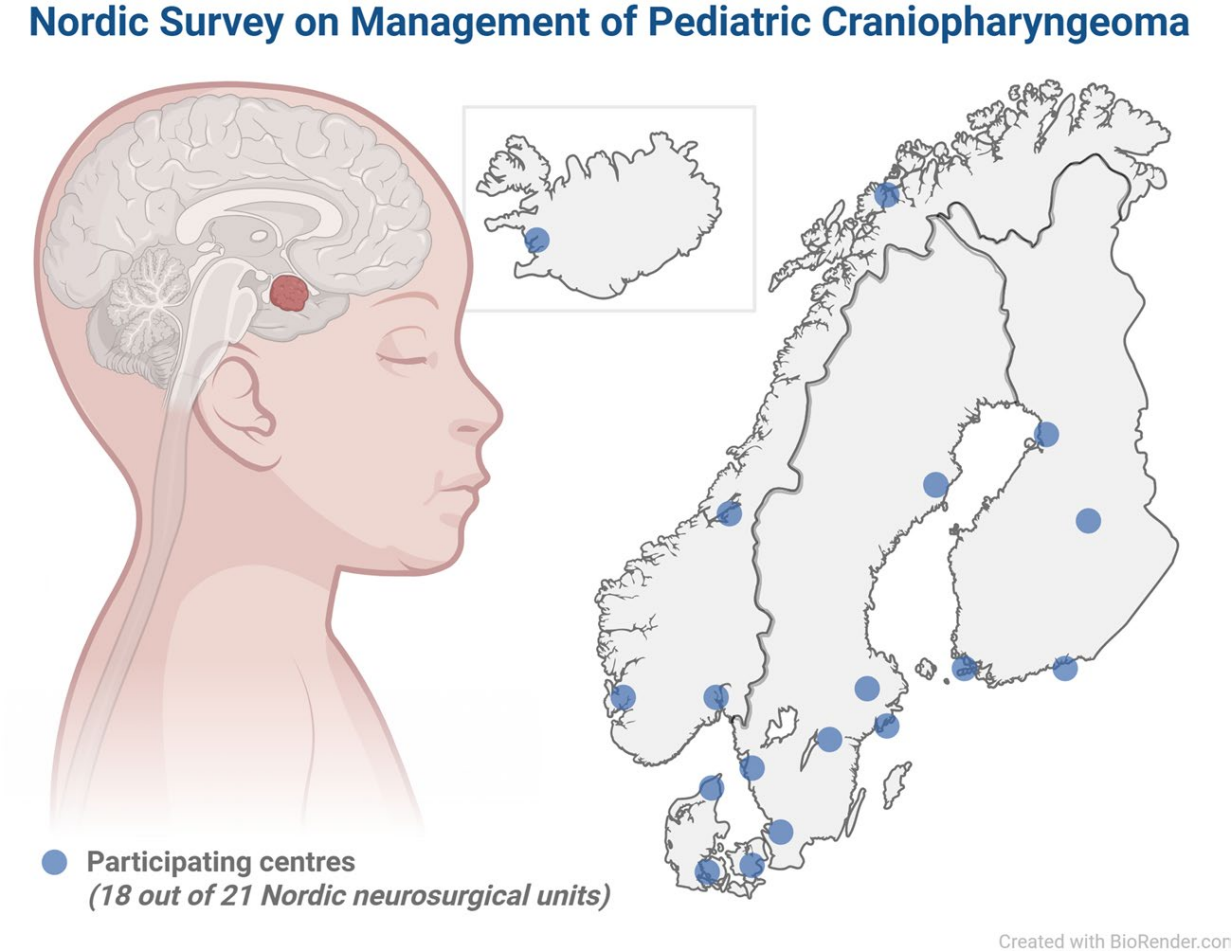
Map of centres participating in the survey

The survey was circulated to all existing 21 academic neurosurgical centres in five Nordic countries: Denmark (*n* = 4), Finland (*n* = 5), Iceland (*n* = 1), Norway (*n* = 4), and Sweden (*n* = 7), as shown in Fig. 1. In total, responses were received from 18 out of 21 centres, giving a response rate of 86%.

### Organization and expertise

Primary referral area (as reported by the centres themselves) varied between 0.45–3.2 million inhabitants of all ages (mean 1.62 million). The estimated numbers of pediatric neurosurgical procedures performed per year, the number of new cases of pediatric CP per year, and the number of all pediatric CP cases treated during the last 20 years (2004–2023) are given in Table 1.

**Table 1:** Self-reported estimated data from responding centres illustrating their exposure to pediatric neurosurgical cases and pediatric CP in particular

		*n* (%)
No. of pediatric neurosurgical procedures/year	<50	7 (39)
	50–100	5 (28)
	100–250	5 (28)
	>250	1 (6)
No. of new cases of pediatric CP/year	0–1	12 (67)
	2–5	6 (33)
	>5	-
No. of cases of pediatric CP during the last 20 years	median 17.5 (range 5–27)	

A dedicated pediatric neurosurgeon was available in 15 centres (83%), with a median of three subspecialists per department (range 1–5). However, only four (22%) responders considered themselves dedicated pediatric neurosurgeons (i.e., dealing mostly with pediatric patients), while 10 (56%) rather a “mixed” pediatric/adult neurosurgeon (i.e., not selected practice), whereas four (22%) described themselves as general neurosurgeons, dealing mostly with adult patients but taking care of pediatric patients when presenting at the department.

**Fig. 2:**
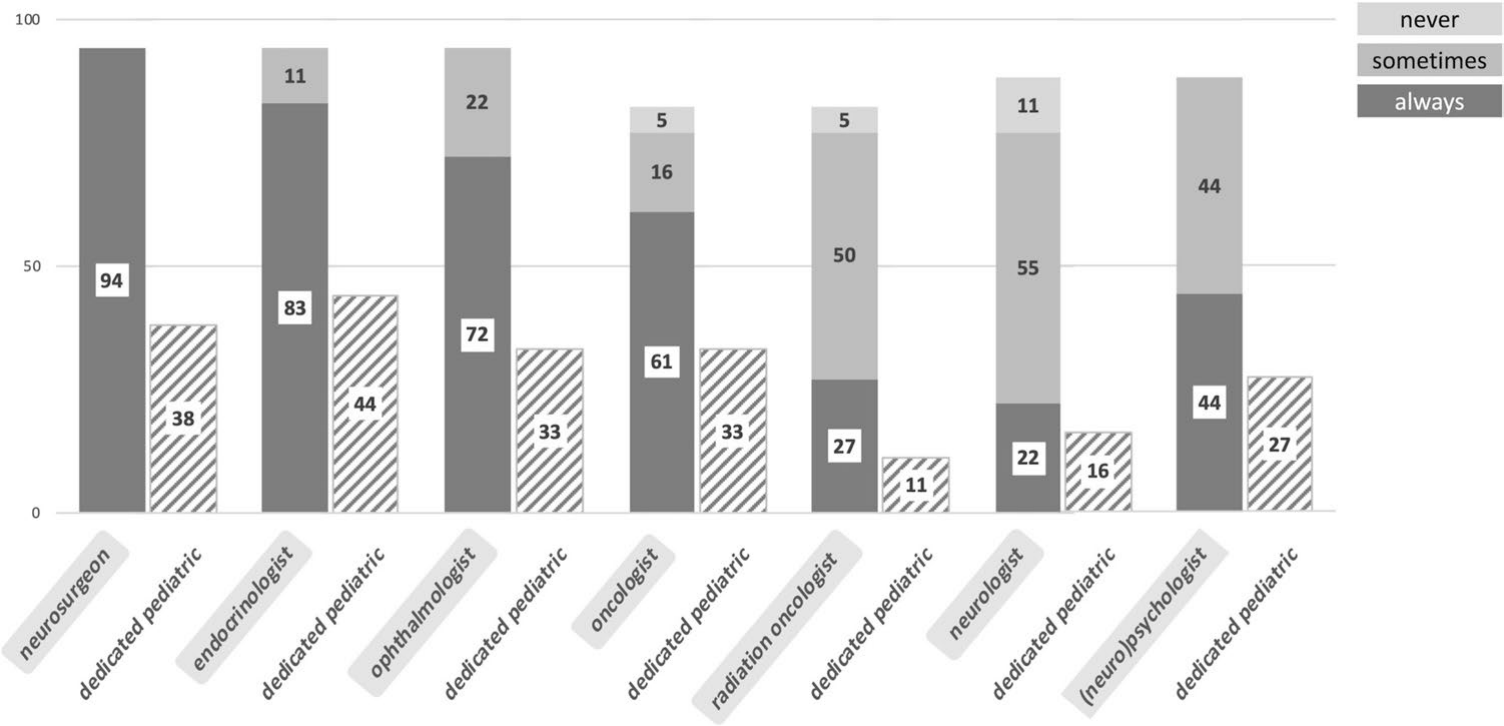
Specialists involved in the treatment of pediatric CP at responding institutions (*n* = 18). Values are given as percentages of replies (“always” in dark grey, “sometimes” in bright grey, and “never” in the brightest grey columns, respectively), as well as the percentage of dedicated pediatric subspecialists within the respective specialties (stripped column). Other mentioned specialists were a gamma knife neurosurgeon, skullbase/pituitary surgeon, ENT surgeon, and pediatrician

According to the replies, 13 (72%) responders/centres treat pediatric CP at their own institution, whereas two (11%) refer these cases to another specialized centre (in both cases due to an administrative decision). The two remaining respondents reported that children with CP were treated both at their own institutions and at other specialized centres—the latter in these cases for intracystic treatment and due to the lack of experience with pediatric CP, respectively. None of the centres reported a lack of another relevant, non-neurosurgical expertise (pediatric endocrinology, ophthalmology, etc.) at their institutions. Medical specialists involved in the treatment of pediatric CP at responding institutions are listed in Fig. 2.

### Treatment decisions and strategies

Newly diagnosed children with CP were reported to be discussed in MDT meetings at all responding centres: in 69% a pediatric neurooncological MDT, while in 13% a “pituitary” MDT, and not specified in the remaining replies. Here, however, a pediatric neurosurgeon is most often responsible for the final decision-making (56%), followed by a pediatric oncologist (38%) or pediatric endocrinologist (13%).

We then asked specifically what diagnostic investigations the responders would require in case of an *incidental finding* of a CP: the result was endocrinology in 100% of replies (always) and ophthalmology in 94% (always, and sometimes in 6%), while neurology always 44% and sometimes 50%, respectively. Then, we also asked what their treatment strategy would be chosen in different clinical and radiological situations. The replies are summarized in Table 2.

**Table 2:** Question: in case of an incidental finding of a CP, what would be your first-line strategy

			Observation (initial)n (%)	Resectionn (%)	Cyst tapping (if cystic tumor)	Other replies(specify)
*Clinic *	*No symptoms at all*		14 (88)	2 (13)	-	a
	*Headache*		4 (25)	7 (44)	6 (38)	a
	*Endocrinological disturbances*		5 (31)	8 (50)	5 (31)	a,c
	*Ophthalmological disturbances*		-	13 (81)	7 (44)	a,c
*MRI*	*Intrasellar, intact chiasm*		12 (75)	4 (25)	-	a,d
	*Suprasellar, intact chiasm*		10 (63)	5 (31)	1 (6)	a,d
	*Suprasellar, affection of chiasm*		1 (6)	11 (69)	3 (31)	a,d,e
	*Intraventricular*		4 (25)	9 (56%)	3 (19)	a,d,e,f
	*Hypothalamus involvement *	*Puget 0*	6 (38)	6 (38)	2 (13)	a,d
		*Puget 1 *	4 (25)	8 (50)	4 (25)	a,d
		*Puget 2*	5 (31)	7 (44)	4 (25)	a,d

^a^ Patient specific treatment choice based on comprehensive evaluation of the case

^b^ Substitution therapy

^c^ Surgery should be considered

^d^ Discuss with experienced centre

^e^ Biopsy, shunt

^f^ Treatment of hydrocephalus if present

In case of MRI finding of *hydrocephalus in an incidental case* of CP, the responders would opt for observation (if asymptomatic) in 5 (31%), while endoscopic 3rd ventriculostomy (if feasible) was mentioned by 7 (44%; with biopsy in 1 case), VP shunt by 5 (31%), tumor resection by 8 (50%), and other measures by 1 (6%; fenestration of the cysts) out of 16 respondents.

### Observation

In case of initial *observation* of a CP, the responders would recommend an interval to the first follow-up MRI as follows: 3 months (*n* = 10; 63%), 6 months (*n* = 4; 25%), and both 3 and 6 months (*n* = 2; 13%), where in one reply “depending on size and proximity to chiasm”. None of the options “12 months”, “other”, or “only when new symptoms occur” was chosen by any of the responders.

Consequently, in case of stable finding between the first two MRIs, the proposed interval for further follow-up MRI would be 3 months (*n* = 4; 25%), 6 months (*n* = 6; 38%), 12 months (*n* = 4; 25%), and 3 and 6 months (*n* = 2; 13%), where in one reply “depending on size and proximity to chiasm”. No one responded “only when new symptoms occur”.

### Treatment

In case of indication for treatment of a newly diagnosed CP, 8 responders (53%) would consider a degree of hypothalamic involvement (according to Puget classification) as a significant factor for their choice of the treatment strategy, whereas 4 (27%) would not, and 3 (20%) did not know (out of total 15 replies).

Table 3 shows what would be the responders’ first choice when selecting a treatment strategy for a newly diagnosed CP.

**Table 3:** Question:if you find indication for treatment of a newly diagnosed CP, your first choice would be

	Surgery		Cyst aspiration *n* (%)	Radiotherapy *n* (%)	Radiosurgery *n* (%)	Other replies (specify)
	*Transcranial n* (%)	*Transsphenoidal* *n* (%)				
Puget 0	4 (25)	7 (44)	2 (13)	-	0	a
Puget 1	7 (44)	4 (25)	2 (13)	-	1 (6)	a,b
Puget 2	8 (50)	2 (13)	3 (19)	-	2 (13)	a
regardless	3 (19)	2 (13)	-	-	-	a,c,d

^a^ Surgery: depends not on Puget alone, but sellar/suprasellar tumor involvement/surgical anatomy

^b^ Depending on and size of sphenoidal sinus

^c^ Transsphenoidal: sometimes dependent on location, invasion and location of anatomical structures

^d^ Patient specific treatment choice related to radiological findings

### Surgery

In case of indication for surgical resection, one responder (6%) would primarily choose a transcranial and two (13%) an endonasal endoscopic approach, whereas 13 (81%) would decide depending on anatomy and surgical feasibility. For replies regarding the competency for the choice of the best surgical approach in each case, see Table 4.

**Table 4:** Question: for the choice of the best surgical approach in each individual case, you would (15 responses)

		*n* (%)
Consider the surgical approach and strategy self		3 (20)
Consult “adult” colleagues who*	Do endonasal surgery at their departments	7 (47)
	Do transcranial approaches for CP more frequently	1 (7)
	For both reasons	1 (7)
(*of these responders, 6 (40%) would in addition consider the approach and strategy self)		
Consult colleagues at another institution		3 (20)

### Lower age limit for endonasal approaches

In cases where an endonasal approach appears the best surgical option, 7 responders (54% of 13 valid responses) replied that the lower age limit for feasibility of such an approach would in their practice be from 1–2 to 13 years, whereas 3 (23%) did not see any lower limit. Two responders (15%) specified that the presence of a sphenoid sinus would influence their consideration of the feasibility of performing an endoscopic approach. Lastly, one responder (8%) stated that an ear-nose-throat (ENT) specialist would be consulted.

### Intervention strategy and competency

We then asked the responders about their main priority when planning and executing first-time neurosurgical intervention for pediatric CP. The instruction was to select only one option, to reflect the responder’s first priority: out of 13 valid responses, 12 (92%) pointed at “maximal safe resection” and the remaining one at “satisfactory decompression of optical nerves/tract”. Other offered options (gross total resection, draining the cyst in order to alleviate the mass effect, restoration of CSF pathways—i.e., treatment of hydrocephalus, if present) were not chosen by anyone. Responses from three other responders were excluded, as they contained multiple option replies, despite being instructed to choose only one.

Finally, we asked whether the responders felt—as pediatric neurosurgeons—competent and experienced enough to treat CP in children: 13 (81%) replied “yes, in all cases”, 3 (19%) replied “only in some cases”, while no one replied “no, in no cases”. Four (25%) of the responders specified the lack of expertise in (extended) endonasal approaches and limited surgical experience with CP as the main reasons for limited competency at their respective centres.

### Follow-up routines

**Fig. 3:**
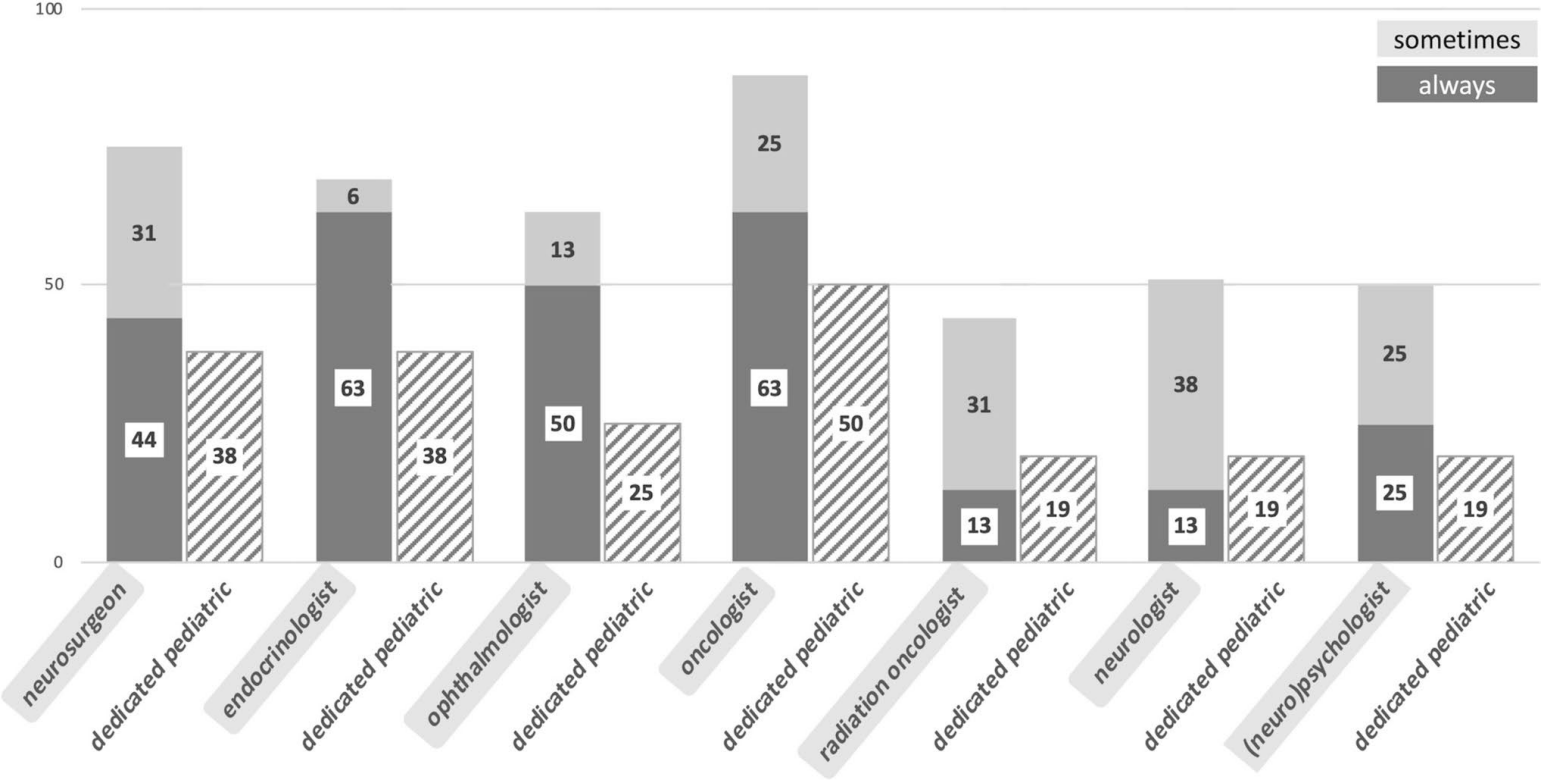
A graph showing which specialists are primarily responsible for follow-up of pediatric patients with CP at the responders` institutions, in percentage of replies (“always” in dark grey and “sometimes” in bright grey columns, respectively), as well as the percentage of dedicated pediatric specialists within the respective specialties (stripped column)

Figure 3 shows which specialists are primarily responsible for follow-up of pediatric patients with CP at the responders` institutions. Here, it appears that endocrinologists (63%), oncologists (63%), ophthalmologists (50%), and neurosurgeons (44%), respectively, are always primarily responsible. However, the proportion of dedicated pediatric specialists was highest for oncologists (50%).

According to the responses, pediatric patients with CP would typically be followed and their MRI controls reviewed by the MDT at all 16 responding institutions: by MDT for oncology in 14 (88%), out of which 9 specifically pediatric, and by MDT for endocrinology (“pituitary-meeting”) in 3 (19%), out of which one specifically pediatric. Two responders claimed that both MDT for oncology and endocrinology would be involved at their institutions, and one responder did not provide any specific answer.

According to the responders’ replies, the biggest challenge during follow-up of children treated for CP in the short term is the endocrinological morbidity (69% of responses), while tumor recurrence was reported by all responders (100%) as major long-term challenge, followed by reduced quality of life (QoL) due to CP and its treatment (81%), hypothalamic obesity (75%) and endocrinological morbidity (56%) (Table 5).

**Table 5:** Question:In your opinion and experience, what are the biggest challenges during the follow-up (either short-term or long-term) of children treated for CP *(multiple choices apply)*

	Short-term*n* (%)	Long-term*n* (%)	Short- & long-term*n* (%)
Tumor recurrences	2 (13)	16 (100)	2 (13)
Endocrinological morbidity	11 (69)	9 (56)	6 (38)
Hypothalamic obesity	3 (19)	12 (75)	3 (19)
Ophthalmological consequences	6 (38)	8 (50)	4 (25)
Post-radiation complications	1 (6)	6 (38)	-
Psychosocial problems, school attendance	3 (19)	8 (50)	2 (13)
Reduced QoL due to CP and its treatment	6 (38)	13 (81)	3 (19)

Finally, we asked how the responders would agree/disagree on a few different statements regarding pediatric CP and its treatment. The responses are summarized in Table 6.

**Table 6:** Question: how would you agree/disagree on following statements

	Absolutely agree*n* (%)	Rather agree*n* (%)	Ambivalent/don’t know*n* (%)	Rather disagree*n* (%)	Absolutely disagree*n* (%)
*Childhood-onset CP requires life-long follow-up*^1^	13 (87)	1 (7)	1 (7)	-	-
*Treatment of pediatric CP should be centralized to centres with and experienced and competent MDT*^2^	7 (50)	5 (36)	1 (7)	1 (7)	-
*Only neurosurgical treatment of CP should be centralized, not necessarily the other specialties*^1^	1 (7)	1 (7)	3 (20)	9 (60)	1 (7)
*I am willing to refer new cases to such centres, if they exist or will be established in our country*^2^	4 (29)	4 (29)	4 (29)	2 (14)	-

Numbers of valid responses: ^1^*n*=15; ^2^*n*=14

## Discussion

The results from the survey conducted among pediatric neurosurgeons from 18 neurosurgical departments in five Nordic countries show that in this part of Europe, characterized by stable but small populations, the case load is very limited: in 61% of centres, only 0–1 new cases of pediatric CP present and are treated per year. Moreover, surgery in children with CP is not performed at all responding institutions, as, for instance in Denmark, the neurosurgical treatment of pediatric CP is centralized to one centre. Several similarities and differences in management were reported between centres: while for instance all centres discuss their cases at MDT meetings before surgery and during the follow-up, management strategies including surgical indications, goals, and approaches may vary among responding centres.

The observed variations in the practice of management of pediatric CP may, of course, be a result of low case volumes. In absolute numbers, the results of this survey cannot be interpreted with any statistical significance. Probably only more populated European countries like the United Kingdom, France, Spain, Italy, Germany, Poland, or Russia would have patient populations (including pediatric CP) large enough to give any significant answers to the questions given in the present survey. However, this survey gives valuable insights that can be applicable in many other European countries of population size similar to the Nordic countries.

The results of the survey suggest that low case exposure at each department is the main challenge when building up competency for the treatment of this vulnerable patient group. In general, a higher case load is favorable in order to achieve competency, experience, and ultimately better results of treatment of seldom-seen pathologies with unique surgical challenges like CP in children. One option is centralization to a very few or, in the case of the Nordic countries, perhaps one institution in each country, providing special competency in the treatment of pediatric CP. One single centre for the whole of Scandinavia would probably not be practically feasible due to geographical challenges, but also language and cultural differences. In the European context, the model of one national centre for pediatric oncology (including CP) has proved successful, for instance in the Netherlands [10]. However, the attitude to such a solution is still ambivalent among responders to the present survey: while the majority of them (75%) were seemingly in favor, 12% of respondents were not, and only 50% stated that they would be willing to refer new cases to other centres with special competency. This is an interesting paradox, probably reflecting the fact that although most responding pediatric neurosurgeons find centralization reasonable in the case of pediatric CP, they may not agree on which centres in their respective countries should have this role. Given the complexity in the treatment and follow-up of pediatric CP, it remains also an important question whether only neurosurgical treatment should be centralized, or all aspects of multidisciplinary care of this disease, including pediatric endocrinology, oncology, neuropsychology, etc. Although the latter format is perhaps most ideal, it may be practically more difficult to execute in countries with scarcely distributed population and large geographical distances (Norway, Sweden, Finland) than in smaller countries with more dense population (Denmark, Netherlands).

In this context, it is important to remember that surgery is only a part of the complex management and follow-up of CP. Particularly from the patients' perspective, adequate care for disease- and treatment-related morbidity in CP, such as acquired hypothalamic dysfunction [11], fatigue or psychosocial health [12], is equally important. In one previous survey, the patients and their families expressed preference for treatment that would improve the quality of life rather than decrease the risk of recurrence, and identified endocrine issues as having the greatest impact on this quality [13]. Obviously, such care can be improved only if delivered by experts with a holistic view of the patient in a multidisciplinary setting and with a focus on quality of life [14].

However, a potential discussion regarding further centralization of neurosurgical care for children with CP should be based on robust evidence, including a clear understanding of how variations in management and case volume may influence the outcomes. Such evidence should be obtained from dedicated multicenter studies, for which the Nordic countries are particularly well-positioned due to similar practice and organization of health care.

Despite differences noticed in this survey, there are to date no known significant differences in the overall survival of patients with childhood-onset CP among different Nordic countries. While this particular fact can be verified by data from respective national health registries, it is less clear whether varying surgical exposure and experience may result in different morbidity related to neurosurgical interventions. Optimally, there should be established national registries – or perhaps a common Nordic one – for children presenting and treated for CP, where such data could be prospectively collected. Similarly, future investigations should focus on highlighting which observed differences noticed in this survey are most likely to influence the clinical outcomes: specifically, e.g. surgical approach, surgeon´s consideration of hypothalamic involvement, use of endonasal technique in pediatric patients etc. In this fashion, it would also be possible to distinguish between differences in management practice that are clinically acceptable from those that could potentially affect morbidity, functional outcomes, and long-term quality of life after treatment.

### Limitations

Although we did not receive replies from three institutions (14%), at least two of them reportedly do not treat children with CP at all, and their responses would therefore hardly influence the overall results. The design of the survey may also be discussed in some details, as would always be the case in a qualitative study like this one. We also noticed that for one question of the survey (no. 22), some responders chose multiple replies instead of only a single one (despite being specifically instructed). Together with the fact that not all questions were answered by all responders, it could negatively influence the validity of these particular replies.

## Conclusion

The practice of neurosurgical management of pediatric CP in the Nordic countries is characterized by relatively low case volumes, differing management strategies, as well as varying degrees of centralization and collaboration between pediatric and adult neurosurgeons. Although some practical aspects may be unique for healthcare practiced in the Nordic countries, the results of this survey and, in particular, the embedded discussion of centralization – either restricted to surgery or including complete management of pediatric CP – seem relevant and may provide useful insights also to neurosurgeons outside of the Nordics.

## Supplementary Information

Below is the link to the electronic supplementary material.

ESM 1 Supplementary Material 1 (PDF 144 KB)

## Data Availability

No datasets were generated or analysed during the current study.
