# Supplementary material for: Nordic survey on practice of neurosurgical management of craniopharyngioma in children
Source: Childs Nerv Syst. 2026 Feb 4;42(1):59. doi: 10.1007/s00381-026-07153-8 (PMC12872692; doi:10.1007/s00381-026-07153-8)
Supplement: Supplementary file 1 — Supplementary Material 1 (PDF 144 KB) [file 381_2026_7153_MOESM1_ESM.pdf]

Nordic survey on treatment practice of childhood-onset craniopharyngioma.

Craniopharyngiomas (CP) are rare, benign tumours, yet with tendency to aggressive growth into critical neural structures. This feature, along with significant morbidity associated with treatment, make the choice of optimal treatment strategy difficult, particularly in children.

Although there has been tendency towards less aggressive surgical approaches in children with CP during the recent two decades, the practice of treatment still differs between centres.

The purpose of this survey is to gain overview on the current standard of practice among the neurosurgical practice in five Nordic countries (Denmark, Finland, Iceland, Norway, Sweden). The public healthcare systems in all these countries are very similar, and treatment of difficult pathologies like CP occurs at a relatively few neurosurgical centres. Together with generally stable populations and good follow-up routines in Nordic countries, it allows for a relevant comparison of treatment strategies between centres.

It will be highly appreciated if you – on behalf of pediatric neurosurgeons at your institution – can fill this survey and return it to radek.fric@medisin.uio.no no later than by April 1, 2025. Neither your name nor the name of your institution will appear in the analysis of the survey results. All replies in this survey will be treated confidentially.

You will be informed about survey results by our working group, before the results will be drafted as an article, in line with the plan suggested in our last meeting of the Nordic Pediatric Neurosurgical Network (NPNN) in Copenhagen in the end of September 2024.

Thank you for your kind cooperation!

Radek Frič (Oslo), Daniel Nilsson (Göteborg) and Jon Foss-Skiftesvik (København), on behalf of NPNN.

A. Organization and expertise

1. Name of your hospital: ……………………………………………………………………..

2. What is your primary referral area? (approx. population) …………………………………..

3. Does you institution have a dedicated pediatric neurosurgeon?

   ☐ yes ☐ no

   (if yes, how many: ………………..)

4. Do you consider yourself:

   ☐ a dedicated pediatric neurosurgeon (i.e., dealing mostly with pediatric patients)
   ☐ a "mixed" pediatric/adult neurosurgeon (i.e., not selected practice)
   ☐ a general neurosurgeon, dealing mostly with adult patients, but taking care of pediatric patients when they present at the department

5. How many **pediatric neurosurgical procedures** does your department perform a year:

   ☐ <50 ☐ 50-100 ☐ 100-250 ☐ >250

   (if known, please give an exact number from 2024: ……………….)

6. How many **new cases of pediatric CP per year** do you see at your institution:

   ☐ 0-1 ☐ 2-5 ☐ >5

7. If this information is easily available, how many such cases has your institution treated during the last 20 years (2005-2024)?

   ……………………………………………………………………….

8. Do you treat these pediatric cases at your institution or refer them to a specialized centre?

   ☐ treat at own institution ☐ refer to a specialized centre

9. If you refer to a specialized centre, it is due to:

☐ administrative decision (e.g. centralization) taken by medical or health-care authorities
☐ lack of neurosurgical experience/expertise with these particular cases at own institution
☐ lack of another relevant expertise (pediatric endocrinology, ophthalmology etc.)
(please specify: …………………………………………………………………...)
☐ not applicable (I don`t refer to any specialized centre)

10. At your institution, **following specialists are involved** in the treatment of pediatric CP (please ndicate if these are dedicated pediatric specialists):

	(dedicated pediatric)	**always**	**sometimes**	**never**
**neurosurgeon**				
**endocrinologist**				
**ophthalmologist**				
**oncologist**				
**radiation oncologist**				
**neurologist**				
**(neuro)psychologist**				
**others:**				

If you don’t treat children with CP at your institution, please skip the rest of the survey.

B. Treatment decisions and strategies

11. In case of **newly diagnosed pediatric CP**, who takes a decision regarding the treatment strategy at your institution (except of cases requiring acute interventions):

☐ neurosurgeon ☐ pediatric
☐ adult
☐ pediatric ☐ oncologist
☐ endocrinologist
☐ multidisciplinary team (MDT) for
☐ pediatric (neuro) oncology
☐(pediatric) (neuro) endocrinology ("hypophysis-meeting")

12. In case of an **incidental finding** of a CP, would you always recommend these investigations as a part of diagnostic workup?

	**always**	**sometimes**	**never**
**endocrinology**			
**ophthalmology**			
**neurology**			
**other** (specify)			

13. In case of an **incidental finding** of a CP, what would be your first-line strategy:

			**observation** (initial)	**resection**	**cyst tapping** (if cystic tumor)	**other** (specify)
Clinic	*no symptoms at all*					
	headache					
	endocrinological disturbances					
	ophthalmological disturbances					
MRI	*intrasellar, intact chiasma*					
	suprasellar, intact chiasma					
	suprasellar, affection of chiasm					
	intraventricular					
	hypothalamus involvement	*Puget 0**				
		Puget 1				
		Puget 2				

**Puget 0 = no hypothalamic involvement, Puget 1 = tumor abutting or displacing the hypothalamus, Puget 2 = invasion of the hypothalamus by the tumor.*

14. In case of MRI finding of **hydrocephalus in an incidental case of CP**, you would opt for:

☐ observation (as long asymptomatic)

☐ endoscopic 3rd ventriculostomy (if feasible)

☐ VP shunt

☐ tumor resection

☐ other: ……………………………………

15. In case of **observation** of a CP, what would be interval to the first follow-up MRI:

☐ 3 months ☐ 6 months ☐ 12 months ☐ other (………..) ☐ only when new symptoms

16. Consequently, in case of **stable findings between the first two MRIs**, what would be your proposed interval for further follow-up MRI:

☐ every 3 months ☐ every 6 months ☐ every 12 months ☐ other (………..) ☐ only when new symptoms

17. If you find indication for treatment of a newly diagnosed CP, would a **degree of hypothalamic involvement** (according to Puget classification as mentioned above) have any significance for your choice of the treatment strategy:

☐ yes ☐ no ☐ don't know

18. If you find indication for treatment of a newly diagnosed CP, your first choice would be:

	**surgery**		**cyst aspiration**	**radiotherapy**	**radiosurgery**	**other**
	transcranial	transsphenoidal				
**Puget 0**						
**Puget 1**						
**Puget 2**						
**regardless***						

**of hypothalamus involvement*

19. In case of surgery, your **first-line surgical approach** would be:

☐ always transcranial
☐ always endonasal endoscopic
☐ always endonasal microsurgical
☐ dependent on anatomy and surgical feasibility

20. For the **choice of the best surgical approach** in each individual case, you would:

☐ consider the surgical approach and strategy yourself
☐ consult "adult" colleagues who ☐ do endonasal endoscopic surgery at your department
☐ do transcranial approaches for CP more frequently
☐ consult colleagues with such experience at another institution

21. If the **endonasal** (endoscopic/microsurgical) **approach** appears the best surgical option, what is the **lower age limit**, above which you would consider such approach at all? …………………………………………………………

22. What is your **main priority** when planning and executing first-time intervention for pediatric CP (select one option reflecting your **first** priority):

☐ gross total resection
☐ maximal safe resection (even if subtotal/partial)
☐ satisfactory decompression of optical nerves/tract
☐ draining the cyst in order to alleviate the mass effect
☐ restoration of CSF pathways (i.e., treatment of hydrocephalus, if present)

23. Do you feel that your local team is competent and experienced enough to treat CP in children:

☐ yes (in all cases) ☐ only in some cases ☐ no (in any cases)

24. If you cannot answer "yes" to the previous question, it is mainly due to following reasons:

☐ not enough surgical experience with (pediatric) CP in general
☐ lack of expertise/experience in skull base microsurgery (transcranial approaches)
☐ lack of expertise/experience in endonasal (endoscopic/microsurgical) approaches
☐ other (please specify): …………………………………………………

C. Follow-up routines

25. Who of these specialists is **primarily responsible for follow-up** of pediatric patients with CP at your institution? Please indicate if these are dedicated pediatric specialists.

	(dedicated pediatric)	**always**	**sometimes**	**never**
**neurosurgeon**				
**endocrinologist**				
**ophthalmologist**				
**oncologist**				
**radiation oncologist**				
**neurologist**				
**(neuro)psychologist**				
**others:**				

26. Would pediatric patients with CP be typically followed and their MRI controls reviewed in the MDT at your institution?

☐ no
☐ yes ☐ MDT for (neuro) oncology ☐ dedicated pediatric
☐ MDT for endocrinology ("hypophysis-meeting") ☐ dedicated pediatric

27. In your opinion and experience, what are the **biggest issues during the follow-up** (either short-term or long-term) of children treated for CP *(multiple choices apply)*:

	**short-term**	**long-term**
**tumor recurrences**		
**endocrinological morbidity**		
**hypothalamic obesity**		
**ophthalmological consequences**		
**post-radiation complications**		
**psychosocial problems including school attendance**		
**reduced quality of life due to CP and its treatment**		

28. Finally, how would you agree/disagree on following statements:

	**absolutely agree**	**rather agree**	**ambivalent / don't know**	**rather disagree**	**absolutely disagree**
Childhood-onset CP requires life-long follow-up					
Treatment of pediatric CP should be centralized to centres with and experienced and competent MDT					
Only neurosurgical treatment of CP should be centralized, not necessarily the other specialties					
I am willing to refer new cases to such centres, if they exist or will be established in our country					
